# Changes in Nursing Home Use Following Medicaid-Supported Expanded Access to Home- and Community-Based Services for Older Adults With Dementia

**DOI:** 10.1001/jamanetworkopen.2023.22520

**Published:** 2023-07-10

**Authors:** Jordan M. Harrison, Flora Sheng, Raina E. Josberger, Harry H. Liu, Patricia W. Stone, José A. Luchsinger, Andrew W. Dick

**Affiliations:** 1 RAND Corporation, Pittsburgh, Pennsylvania; 2 RAND Corporation, Arlington, Virginia; 3 New York State Department of Health, Albany, New York; 4 Analytica Now LLC, Brookline, Massachusetts; 5 Center for Health Policy, Columbia University School of Nursing, New York, New York; 6 Department of Medicine, Columbia University Irving Medical Center, New York, New York; 7 Department of Epidemiology, Columbia University Irving Medical Center, New York, New York; 8 RAND Corporation, Boston, Massachusetts

## Abstract

**Importance:**

New York State’s Medicaid managed long-term care (MLTC) program expanded access to home- and community-based services, providing an alternative to nursing home care for people with dementia. Between 2012 and 2015, the state implemented mandatory MLTC for dual Medicare and Medicaid enrollees requiring more than 120 days of community-based long-term care.

**Objective:**

To evaluate changes in nursing home use among older adults with dementia following MLTC implementation.

**Design, Setting, and Participants:**

This cohort study used longitudinal data from January 1, 2011, to December 31, 2019, from the Minimum Data Set and Medicare administrative data. The study sample included New York State Medicare beneficiaries 65 years and older with dementia. New York City residents were excluded due to insufficient pre–study period data. Data were analyzed from January 1, 2011, to December 31, 2019.

**Exposure:**

Mandatory MLTC enrollment.

**Main Outcomes and Measures:**

Longitudinal models were used to evaluate changes in annual days of nursing home use following the staggered implementation of MLTC across 13 regions of the state. Two models were estimated: (1) a logistic regression model for any nursing home use in a given year and (2) a linear regression model of total nursing home days, conditional on any nursing home use. Models included annual event-time indicators specified as years until or since MLTC implementation. To capture MLTC effects for dual enrollees relative to non–dual Medicare enrollees, models included interaction terms for dual enrollment and event-time indicators.

**Results:**

This sample included 463 947 Medicare beneficiaries with dementia who lived in New York State between 2011 and 2019 (50.2% younger than 85 years; 64.4% women). Implementation of MLTC was associated with lower odds of nursing home use among dual enrollees, ranging from 8% lower odds 2 years post implementation (adjusted odds ratio, 0.92 [95% CI, 0.86-0.98]) to 24% lower odds 6 years post implementation (adjusted odds ratio, 0.76 [95% CI, 0.69-0.84]). Compared with a scenario of no MLTC, MLTC implementation was associated with an 8% reduction in annual days of nursing home use between 2013 and 2019 (mean, −5.6 [95% CI, −6.1 to −5.1] days per year).

**Conclusions and Relevance:**

The findings of this cohort study suggest that implementation of mandatory MLTC in New York State was associated with less nursing home use among dual enrollees with dementia and that MLTC may help prevent or delay nursing home placement among older adults with dementia.

## Key Points

**Question:**

Is implementation of a mandatory managed long-term care (MLTC) program associated with changes in nursing home use among older adults with dementia?

**Findings:**

In this cohort study of 463 947 Medicare beneficiaries with dementia, implementation of mandatory Medicaid MLTC in New York State between 2012 and 2015 was associated with a decrease in nursing home use among dual enrollees 65 years and older with dementia.

**Meaning:**

These findings suggest that mandatory MLTC may help to prevent or delay nursing home placement among older adults with dementia.

## Teaser

This cohort study uses longitudinal models to evaluate changes in nursing home use among older adults with dementia in New York State following implementation of the Medicaid managed long-term care program (MLTC).

## Introduction

The demand for long-term services and supports (LTSS) in the US has increased dramatically in recent years as the population has aged, driven largely by LTSS needs for people with dementia.^1^ More than 7 million US residents have Alzheimer disease and related dementias, a number projected to double over the next 20 years.^2^ As this trend continues, the current rate of informal caregiving for people with dementia has become unsustainable.^3^ Care of people with dementia is particularly challenging due to the long period of disability and dependence before death, often compounded by behavioral and psychological symptoms such as wandering and agitation.^4,5,6,7^ Caregiving demands create physical, emotional, and financial strain for family caregivers.^8^ As caregiving needs become greater, many people with dementia enter a nursing home.^5^

Medicaid is the country’s largest payer for LTSS, including institutional care in nursing homes as well as home- and community-based services (HCBS). State Medicaid programs play a critical role in addressing the growing challenges with the quality and costs of dementia care. These challenges include long-standing geographic, socioeconomic, and racial and ethnic disparities in the quality of nursing home care and access to HCBS.^9,10,11,12,13,14^ Home- and community-based services encompass a range of health and human services such as personal care services, adult day programs, and care management. Over the past 2 decades, state Medicaid reforms have emphasized LTSS rebalancing from nursing homes to HCBS, allowing more individuals who need assistance with everyday activities to remain in their homes and communities. Home- and community-based services are generally less costly per enrollee than institutional care and more consistent with older adults’ preferences to “age in place.”^15^ However, Medicaid is the sole source of coverage for many HCBS, as these services are not typically covered by private insurance or Medicare.^16^ Further, access to Medicaid HCBS still varies widely within and across states, leading to gaps and inequities in enrollment and use of services.^15^

To streamline delivery of Medicaid LTSS, many states have transitioned from fee-for-service models to managed LTSS (MLTSS) programs. Under MLTSS, managed care plans receive capitated payments to deliver LTSS to enrollees, either as a stand-alone benefit or as part of a comprehensive package of medical care and LTSS.^17^ Older adults are among the largest populations served by MLTSS.^17^ Across the 25 states that have implemented MLTSS, goals include improving quality of care, expanding access to HCBS, improving care coordination and cost efficiency, and increasing consumer choice of services and setting.^18^ By expanding access to HCBS, MLTSS may delay or prevent nursing home placement among older adults. However, evaluations of state MLTSS programs have shown mixed results across programs and populations with regard to changes in nursing home use.^19,20^ It is unclear how transitions to MLTSS have affected setting of care for people with dementia, who typically have much greater reliance on nursing homes compared with long-term care users without dementia.^10^

New York State’s managed long-term care (MLTC) program provides LTSS to individuals who are chronically ill or disabled and wish to remain in their homes and communities.^21^ Managed long-term care was implemented as part of the New York State Medicaid redesign, a series of large-scale reforms to lower spending and improve care coordination. Between 2012 and 2015, the state transitioned from fee-for-service delivery of Medicaid LTSS to the mandatory MLTC model. Managed long-term care was implemented in phases, starting in New York City and gradually expanding to other regions of the state. All individuals older than 21 years who were dually eligible for Medicaid and Medicare and needed community-based long-term care services for more than 120 days were required to enroll.^21^ In a preliminary evaluation of the program, voluntary enrollment in MLTC prior to 2012 was associated with less use of nursing home care and greater use of HCBS compared with Medicaid fee-for-service enrollees.^19^ However, it is unclear to what extent implementation of mandatory MLTC led to changes in setting of care for people with dementia.

The staggered implementation of MLTC by region provides an opportunity to evaluate the association of MLTC with changes in nursing home use. In this retrospective cohort study, we used longitudinal models to evaluate changes in nursing home use among older adults with dementia in New York State following implementation of MLTC. We hypothesized that MLTC was associated with a decrease in annual days of nursing home use among people with dementia.

## Methods

This cohort study was approved with a waiver of consent for the use of deidentified data by the RAND Human Subjects Protection Committee. We followed the Strengthening the Reporting of Observational Studies in Epidemiology (STROBE) reporting guideline.

### Data Sources

We linked individual-level longitudinal data (January 1, 2011, to December 31, 2019) from the Medicare Master Beneficiary Summary File (MBSF) and nursing home assessment data from the Minimum Data Set 3.0. The MBSF annual file contains Medicare beneficiary demographic characteristics and enrollment data, and the chronic conditions segment contains chronic conditions and dates of diagnoses. The Minimum Data Set is a comprehensive, standardized resident assessment tool mandated for use in all federally licensed nursing homes in the US. Assessments are completed on admission (or readmission), quarterly, and any time residents have a change in health status leading to discharge, death, or transfer to another facility.

We used Minimum Data Set assessment data to measure total annual days of long-term care nursing home use among New York State Medicare beneficiaries. We counted all days between the admission date and discharge, transfer, or death date as nursing home days, excluding days outside the nursing home (eg, days in acute care settings).^22^ Rehabilitation stays covered by Medicare were not counted toward total long-term care nursing home days.

We used publicly available data from the New York State Department of Health to identify dates of MLTC implementation by region (ie, the effective date of mandatory MLTC enrollment for dual-eligible Medicaid enrollees requiring more than 120 days of community-based long-term care services) (eTable in Supplement 1). To account for changes in nursing home capacity over time, we used publicly available nursing home bed census data from the New York State Department of Health to calculate nursing home bed capacity per capita by county-year.

### Study Sample

We used annual state codes in the MBSF to identify Medicare beneficiaries 65 years and older who resided in New York State between 2011 and 2019. We excluded individuals who were younger than 65 years in a given year. Individuals who died during the study period were dropped from the sample in subsequent years. We excluded individuals who lived outside New York State at any point during this period. We also excluded New York City residents from our sample due to insufficient pre–study period data for our main analyses, as New York City was the first region to implement MLTC in 2012. We identified annual dual-enrollment status using the MBSF, categorized as zero months of Medicaid enrollment (non–dual enrollment) or 1 to 12 months of Medicaid enrollment (dual enrollment).

To identify individuals with dementia diagnoses, we used the Chronic Conditions Data Warehouse indicator of Alzheimer disease and related disorders or senile dementia reported in the MBSF chronic conditions segment. This variable indicates the date the beneficiary first met claims-based criteria for Alzheimer disease or dementia diagnosis based on *International Classification of Diseases, Ninth Revision*, or *International Statistical Classification of Diseases and Related Health Problems, Tenth Revision*, codes within a 3-year look-back window. We used this variable to create an annual indicator of dementia status, equal to 1 starting in the year of diagnosis. The Chronic Conditions Data Warehouse algorithm has high sensitivity and reasonable specificity for identifying individuals with dementia.^23^

### Statistical Analysis

Data were analyzed from January 1, 2011, to December 31, 2019. We first examined unadjusted trends from 2011 to 2019 in the annual share of Medicare beneficiaries with dementia in New York State who had any long-term care nursing home use. We examined these trends overall and stratified by dual enrollment status.

We estimated longitudinal models to evaluate the association of MLTC with changes in nursing home use among dual enrollees with dementia. We used a 2-part model. First, we used logistic regression to estimate the odds of any long-term care nursing home use in a given year. The unit of analysis was the person-year. Second, we used ordinary least squares regression to estimate total annual days of long-term care nursing home use, conditional on any nursing home use. Models included annual event-time indicators (lead or lag dummies) specified by region as years until or since MLTC implementation. To quantify the association between MLTC and nursing home use for dual enrollees relative to non–dual Medicare enrollees, models included interaction terms for dual enrollment and the annual event-time indicators. Models were adjusted for calendar year and individual characteristics, including age group (65-74, 75-84, and ≥85 years), sex, chronic conditions (heart failure, ischemic heart disease, chronic obstructive pulmonary disease, diabetes, and chronic kidney disease), and Medicare managed care enrollment. Models were also adjusted for county fixed effects and county nursing home bed capacity per capita. Models were weighted by region-year such that every region-year had the same influence on the estimates. Standard errors were clustered at the region level.

We used estimates from the 2 models to calculate mean annual nursing home days in a standardized population under 2 scenarios: (1) all regions implemented MLTC on January 1, 2013, and (2) no regions ever implemented MLTC. We hypothesized that mean estimated annual nursing home days were lower under scenario 1 (MLTC) vs scenario 2 (no MLTC). First, we used estimates from the logit model to estimate odds of any long-term care nursing home use for a standardized population (the 2011 population of New York State Medicare beneficiaries aged ≥65 years with dementia). We then used estimates from the linear model to estimate the number of nursing home days, conditional on any nursing home use. We calculated the product of the predictions from the 2 models for each observation, first under scenario 1 and then scenario 2, to generate estimated annual days of nursing home use for each person in the standardized population under each scenario. We used bootstrap methods with clustered resampling to estimate SEs and 95% CIs for the mean estimated annual nursing home days under each scenario.

Analyses were performed in Stata, version 16 (StataCorp LLC), and SAS, version 9.4 (SAS Institute Inc). For all analyses, we used 2-sided tests with α = .05 to indicate statistical significance.

## Results

Our sample included 463 947 Medicare beneficiaries with dementia who lived in New York State between 2011 and 2019, for a total of 1 645 076 person-years. Table 1 shows beneficiary characteristics by person-year, overall and stratified by long-term care nursing home users and nonusers. Overall, 50.2% of Medicare beneficiaries with dementia were younger than 85 years, 64.4% were women, 35.6% were men, and 39.3% were dually enrolled in Medicaid. In terms of race and ethnicity, 0.1% were American Indian or Alaska Native, 1.4% were Asian or Pacific Islander, 6.5% were Black, 3.9% were Hispanic or Latino, 87.0% were non-Hispanic White, and 1.0% were of other or unknown race or ethnicity. Most beneficiaries (60.3%) had been diagnosed with dementia for less than 5 years. Compared with nonusers, long-term care nursing home users were older (59.3% vs 47.4% were aged ≥85 years), were more likely to be dually enrolled in Medicaid (65.3% vs 32.6%), and had higher prevalence of chronic conditions such as cardiovascular disease and chronic obstructive pulmonary disease.

**Table 1.** Characteristics of Medicare Beneficiaries With Dementia in New York State, Overall and by Long-Term Care Nursing Home Use, 2011 to 2019^a^

Characteristic	Person-years, No. (%)		
	Overall	Non–long-term care nursing home users	Long-term care nursing home users
Total	1 645 076 (100)	1 309 633 (79.6)	335 443 (20.4)
Study year			
2011	183 617 (11.2)	145 349 (11.1)	38 268 (11.4)
2012	182 789 (11.1)	146 601 (11.2)	36 188 (10.8)
2013	180 614 (11.0)	144 351 (11.0)	36 263 (10.8)
2014	177 499 (10.8)	142 268 (10.9)	35 231 (10.5)
2015	178 847 (10.9)	142 358 (10.9)	36 489 (10.9)
2016	182 673 (11.1)	144 933 (11.1)	37 740 (11.3)
2017	185 280 (11.3)	146 568 (11.2)	38 712 (11.5)
2018	186 713 (11.3)	148 066 (11.3)	38 647 (11.5)
2019	187 044 (11.4)	149 139 (11.4)	37 905 (11.3)
Individual characteristics			
Dual enrollment in Medicaid, mo			
0	999 338 (60.7)	882 815 (67.4)	116 523 (34.7)
1-12	645 738 (39.3)	426 818 (32.6)	218 920 (65.3)
Medicare Advantage enrollment, mo			
0	1 393 841 (84.7)	1 130 738 (86.3)	263 103 (78.4)
1-12	251 235 (15.3)	178 895 (13.7)	72 340 (21.6)
Time since dementia diagnosis, y			
<5	992 162 (60.3)	780 925 (59.6)	211 237 (63.0)
≥5	652 914 (39.7)	528 708 (40.4)	124 206 (37.0)
Age group, y			
65-74	257 638 (15.7)	218 767 (16.7)	38 871 (11.6)
75-84	567 768 (34.5)	469 982 (35.9)	97 786 (29.2)
≥85	819 670 (49.8)	620 884 (47.4)	198 786 (59.3)
Sex			
Women	1 060 108 (64.4)	837 405 (63.9)	222 703 (66.4)
Men	584 968 (35.6)	472 228 (36.1)	112 740 (33.6)
Race and ethnicity^b^			
American Indian or Alaska Native	2220 (0.1)	1814 (0.1)	406 (0.1)
Asian or Pacific Islander	23 128 (1.4)	19 886 (1.5)	3242 (1.0)
Black	107 506 (6.5)	85 089 (6.5)	22 417 (6.7)
Hispanic or Latino	64 975 (3.9)	54 524 (4.2)	10 451 (3.1)
Non-Hispanic White	1 430 791 (87.0)	1 134 235 (86.6)	296 556 (88.4)
Other	9730 (0.6)	8360 (0.6)	1370 (0.4)
Unknown	6726 (0.4)	5725 (0.4)	1001 (0.3)
Chronic kidney disease			
Never	885 047 (53.8)	740 197 (56.5)	144 850 (43.2)
Ever	760 029 (46.2)	569 436 (43.5)	190 593 (56.8)
Chronic obstructive pulmonary disease			
Never	964 078 (58.6)	790 913 (60.4)	173 165 (51.6)
Ever	680 998 (41.4)	518 720 (39.6)	162 278 (48.4)
Diabetes			
Never	758 160 (46.1)	610 447 (46.6)	147 713 (44.0)
Ever	886 916 (53.9)	699 186 (53.4)	187 730 (56.0)
Heart failure			
Never	766 495 (46.6)	646 677 (49.4)	119 818 (35.7)
Ever	878 581 (53.4)	662 956 (50.6)	215 625 (64.3)
Ischemic heart disease			
Never	415 916 (25.3)	346 344 (26.4)	69 572 (20.7)
Ever	1 229 160 (74.7)	215 625 (64.2)	265 871 (79.3)
Region^c^			
2	665 846 (40.5)	550 644 (42.0)	115 202 (34.3)
3	101 246 (6.2)	80 745 (6.2)	20 501 (6.1)
4	266 960 (16.2)	203 236 (15.5)	63 724 (19.0)
5	68 721 (4.2)	54 522 (4.2)	14 199 (4.2)
6	80 849 (4.9)	60 096 (4.6)	20 753 (6.2)
7	67 985 (4.1)	53 987 (4.1)	13 998 (4.2)
8	100 127 (6.1)	77 784 (5.9)	22 343 (6.7)
9	18 347 (1.1)	14 240 (1.1)	4107 (1.2)
10	52 098 (3.2)	40 849 (3.1)	11 249 (3.4)
11	95 012 (5.8)	72 524 (5.5)	22 488 (6.7)
12	11 190 (0.7)	8640 (0.7)	2550 (0.8)
13	116 695 (7.1)	92 366 (7.1)	24 329 (7.3)

^a^ Residents of New York City (region 1) are excluded. Long-term care nursing home use excludes rehabilitation stays covered by Medicare. Percentages have been rounded and may not total 100.

^b^ Beneficiary race and ethnicity were identified based on the Research Triangle Institute code in the Master Beneficiary Summary File.

^c^ Region 2 includes Nassau, Suffolk, and Westchester; region 3, Orange and Rockland; region 4, Albany, Erie, Monroe, and Onondaga; region 5, Columbia, Putnam, Sullivan, and Ulster; region 6, Cayuga, Herkimer, Oneida, and Rensselaer; region 7, Greene, Saratoga, Schenectady, and Washington; region 8, Broome, Dutchess, Fulton, Montgomery, and Schoharie; region 9, Delaware and Warren; region 10, Madison, Niagara, and Oswego; region 11, Chenango, Cortland, Genesee, Livingston, Ontario, Orleans, Otsego, Steuben, Tioga, Tompkins, Wayne, and Wyoming; region 12, Cattaraugus; and region 13, Allegany, Chautauqua, Chemung, Clinton, Essex, Franklin, Hamilton, Jefferson, Lewis, Schuyler, Seneca, St Lawrence, and Yates.

**Figure 1.**
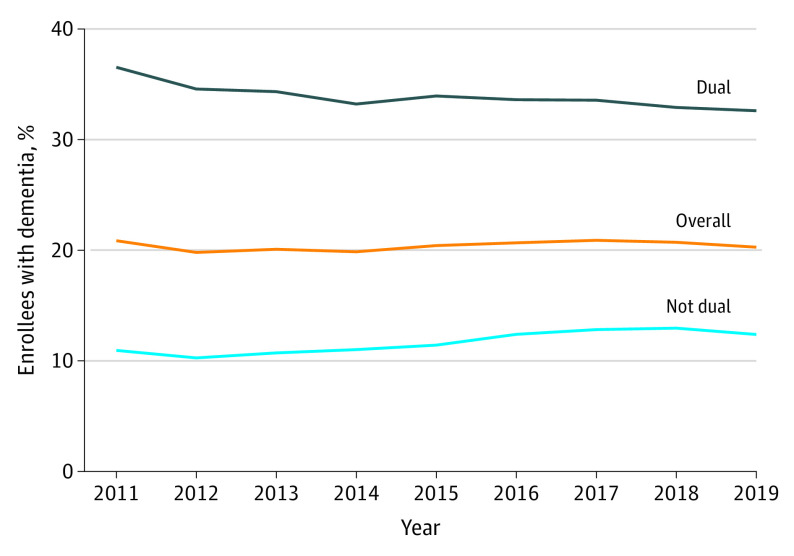
Unadjusted Trends in the Annual Share of New York State Medicare Beneficiaries With Dementia Who Had Any Long-Term Care Nursing Home Use, Overall and by Dual Enrollment Status Data are obtained from January 1, 2011, through December 31, 2019. Residents of New York City are excluded.

Between 2011 and 2019, approximately one-third of dual enrollees with dementia resided in nursing homes during a given year, compared with 10% to 13% of non–dual Medicare enrollees with dementia (Figure 1). The share of dual enrollees with dementia who had any long-term care nursing home use declined from 36.5% in 2011 to 32.6% in 2019. By comparison, the share of non–dual enrollees with dementia with any long-term care nursing home use increased from 10.9% in 2011 to 12.4% in 2019.

Implementation of MLTC was associated with lower odds of long-term care nursing home use among dual enrollees with dementia, ranging from 8% lower odds 2 years post implementation (adjusted odds ratio, 0.92 [95% CI, 0.86-0.98]) to 24% lower odds 6 years post implementation (adjusted odds ratio, 0.76 [95% CI, 0.69-0.84]) (Table 2). Conditional on any long-term care nursing home use during the year, MLTC was associated with small increases in total days of nursing home use in years 3 and 6 post implementation.

**Table 2.** Event-Time Models Estimating the Association of MLTC Implementation With Long-Term Care Nursing Home Use Among Medicare Beneficiaries With Dementia^a^

	Model 1 logit: odds of any long-term care nursing home days, AOR (95% CI) (n = 1 645 076)	Model 2 OLS: mean long-term care nursing home days, coefficient (95% CI) (n = 335 443)
Years since MLTC × dual enrollment in Medicaid		
Dual × event-time <0	1 [Reference]	1 [Reference]
Dual × event-time 1	0.95 (0.89 to 1.02)	−0.12 (−5.99 to 5.76)
Dual × event-time 2	0.92 (0.86 to 0.98)	0.18 (−6.44 to 6.80)
Dual × event-time 3	0.86 (0.82 to 0.90)	7.13 (0.91 to 13.34)
Dual × event-time 4	0.79 (0.75 to 0.83)	3.72 (−2.55 to 10.00)
Dual × event-time 5	0.79 (0.74 to 0.85)	7.41 (−1.57 to 16.39)
Dual × event-time ≥6	0.76 (0.69 to 0.84)	7.28 (0.86 to 13.70)
Dual enrollment in Medicaid, mo		
0	1 [Reference]	1 [Reference]
1-12	4.52 (4.28 to 4.77)	105.63 (98.08 to 113.18)
Time since MLTC, y		
Event-time <0	1 [Reference]	1 [Reference]
Event-time 1	1.05 (1.00 to 1.10)	−4.88 (−13.95 to 4.20)
Event-time 2	1.11 (1.01 to 1.22)	−9.93 (−22.89 to 3.03)
Event-time 3	1.17 (1.00 to 1.36)	−17.04 (−36.56 to 2.47)
Event-time 4	1.22 (1.01 to 1.47)	−20.09 (−39.00 to −1.17)
Event-time 5	1.22 (1.00 to 1.49)	−25.34 (−51.83 to 1.14)
Event-time ≥6	1.26 (0.97 to 1.65)	−29.74 (−60.91 to 1.44)
Year		
2011	1 [Reference]	1 [Reference]
2012	0.93 (0.90 to 0.96)	−2.01 (−4.61 to 0.59)
2013	0.92 (0.89 to 0.96)	−3.25 (−8.91 to 2.41)
2014	0.87 (0.80 to 0.95)	0.05 (−9.05 to 9.14)
2015	0.91 (0.80 to 1.03)	−1.59 (−14.58 to 11.39)
2016	0.92 (0.79 to 1.07)	−0.21 (−18.80 to 18.37)
2017	0.92 (0.77 to 1.10)	0.85 (−19.25 to 20.95)
2018	0.91 (0.75 to 1.11)	1.47 (−23.01 to 25.95)
2019	0.86 (0.65 to 1.14)	5.82 (−23.22 to 34.86)

Abbreviations: AOR, adjusted odds ratio; MLTC, managed long-term care; OLS, ordinary least squares.

^a^ Residents of New York City are excluded from these analyses. Estimates in model 2 are conditional on any long-term care nursing home use during the year. Models are adjusted for individual characteristics (age, sex, chronic conditions, Medicare managed care enrollment), county fixed effects, and county nursing home bed capacity per capita. Estimates are weighted by region and SEs are clustered at the region level.

**Figure 2.**
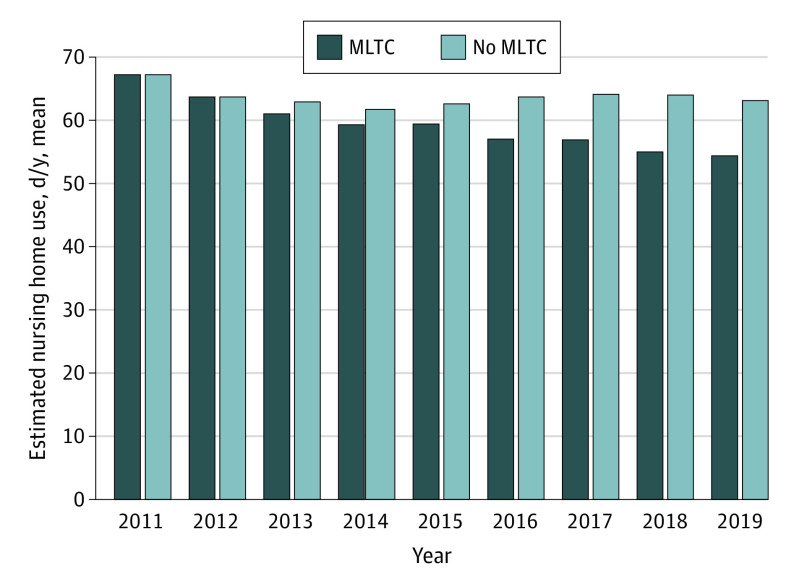
Estimated Mean Annual Long-Term Care Nursing Home Days Among Dual Enrollees With Dementia Estimated annual days of long-term care nursing home use from January 1, 2011, through December 31, 2019, were generated for 2 scenarios, holding 2011 population characteristics constant: all regions implemented managed long-term care (MLTC) on January 1, 2013; and no regions ever implemented MLTC (no MLTC). Residents of New York City are excluded from these analyses.

Figure 2 shows changes over time in estimated annual days of nursing home use among dual enrollees with dementia under the MLTC and non-MLTC scenarios. Compared with a scenario of no MLTC, MLTC implementation across all regions was associated with an 8% reduction in annual days of nursing home use between 2013 and 2019 (mean, −5.6 [95% CI, −6.1 to −5.1] days per year).

## Discussion

As many states implement and expand Medicaid MLTSS, these programs are playing a growing role in long-term care delivery. However, to date, evidence is limited regarding the implications of MLTSS for nursing home use, and to our knowledge, no previous studies have rigorously evaluated the outcomes of MLTSS for people with dementia. Consistent with previous findings,^10^ our cohort study found that older adults with dementia in New York State had high reliance on nursing homes. Over 30% of dual enrollees with dementia resided in nursing homes during a given year. Implementation of MLTC was associated with decreased nursing home use among dually enrolled older adults with dementia, suggesting that MLTC may help to prevent or delay nursing home placement for this population.

In a previous evaluation,^24^ New York’s rollout of mandatory MLTC was associated with a large increase in MLTC enrollment from 2012 to 2018, with no evidence of adverse outcomes such as increased emergency department visits, falls, or avoidable hospitalizations. These findings suggest MLTC plans were able to accommodate increased enrollment without compromising quality of care. Consumer satisfaction measures declined slightly during the first years of the mandate, but the only statistically significant change associated with MLTC rollout was decreased satisfaction with care managers.^24^

Further work is needed to understand the observed decrease in nursing home use associated with MLTC, including the extent to which HCBS provided through MLTC may substitute for nursing home care. Greater access to and use of New York’s Consumer-Directed Personal Assistance Services (CDPAS), which were provided through MLTC plans, may have contributed to lower nursing home use.^25^ Designed to divert enrollees from nursing home care to less costly HCBS, CDPAS allows individuals to direct their own services by hiring caregivers, often family members. Consumer-Directed Personal Assistance Services also provide access to services such as personal care workers and medical daycare that may allow individuals who would otherwise require nursing home care to remain in the community. Enrollment in CDPAS increased 88% from fiscal years 2014 to 2019.^26^

Though MLTC was associated with lower odds of nursing home use, we found that conditional on any nursing home use, MLTC was associated with small increases in total nursing home days. This finding may reflect a selection effect that led to increased acuity of nursing home users following implementation of MLTC (ie, if the reduction in any nursing home use occurred largely among those with lower caregiving needs). Other aspects of MLTC may have contributed to changes in nursing home use during this period, including transition of nursing home populations to MLTC in 2015. Notably, New York has since limited the MLTC nursing home benefit to 3 months of permanent placement for the partial capitation program.

Findings with regard to MLTSS outcomes in other states have been mixed. In a multistate evaluation,^20^ MLTSS enrollees had better self-reported access to care, experiences of care, and quality of life compared with Medicaid fee-for-service enrollees. However, there were no clear patterns with regard to use of nursing homes, HCBS, or acute care; findings varied widely across states and populations.^20^ Though MLTSS has potential to improve the delivery of LTSS to better meet consumer needs and preferences, it may result in adverse outcomes for enrollees if the scope and generosity of HCBS provided by MLTSS plans are inadequate.^20^ More than half of community-dwelling older adults with dementia report experiencing 1 or more adverse consequences due to unmet LTSS needs,^27^ and evidence from previous studies suggests older adults receiving Medicaid HCBS have higher rates of hospital admissions relative to those residing in nursing homes.^28,29^ Future evaluations of MLTSS programs should examine the type and scope of services that meet the needs of people with dementia and their informal caregivers, in addition to comparing outcomes for enrollees with dementia across different settings of care. In addition, further evaluation is needed to understand the impact of MLTSS on geographic, socioeconomic, and racial and ethnic disparities in long-term care quality and access for people with dementia.

### Limitations

This study has some limitations. Our goal in designing this approach was to limit threats to causal inference in identifying the effects of mandatory MLTC on nursing home use. However, our findings should be interpreted in the context of the following limitations. Our analyses excluded New York City, as we did not have sufficient pre–study period data for this region to evaluate changes from pre-MLTC to post-MLTC. Implicit in models such as ours is an assumption that policy effects are homogeneous across regions. These assumptions are likely violated due to heterogeneity of policy implementation across counties and potential spillover effects from counties with earlier implementation of MLTC. Our analyses focus on the entire population of dual enrollees with dementia, as we did not have access to measures of cognitive status or self-care ability to identify the population of individuals at greatest risk for nursing home placement. We relied on Medicare claims to identify individuals with dementia. In addition, we cannot fully account for other policies or contextual factors that may have influenced nursing home use during this period, including other aspects of the New York State Medicaid Redesign. We are unable to identify the use of specific HCBS and their potential contribution to changes in nursing home use. We are, however, able to characterize how MLTC implementation was associated with changes in nursing home use with models that substantially limit threats to causal inference. Finally, generalizability of our findings to other states may be limited by differences across states in MLTSS and Medicaid eligibility criteria and program design features.

## Conclusions

In the context of growing concerns about the quality and cost of long-term care, MLTSS offers a potential solution to manage costs and improve consumer choice of services and setting. The findings of this cohort study suggest that implementation of New York State’s MLTC program was associated with decreased nursing home use among dual enrollees with dementia. Further work is needed to evaluate how transitions to MLTSS have affected the setting and quality of care for people living with dementia, including the extent to which HCBS may substitute for nursing home care.

## Notes

Supplement 1. **eTable.** Managed Long-term Care Mandate Rollout Dates

Supplement 2.
Data Sharing Statement
