# Supplementary material for: Changes in Nursing Home Use Following Medicaid-Supported Expanded Access to Home- and Community-Based Services for Older Adults With Dementia
Source: JAMA Netw Open. 2023 Jul 10;6(7):e2322520. doi: 10.1001/jamanetworkopen.2023.22520 (PMC10334251; doi:10.1001/jamanetworkopen.2023.22520)
Supplement: Supplement 1. — eTable. Managed Long-term Care Mandate Rollout Dates [file jamanetwopen-e2322520-s001.pdf]

Supplementary Online Content

Harrison JM, Sheng F, Josberger RE, et al. Changes in nursing home use following Medicaid-supported expanded access to home- and community-based services for older adults with dementia. *JAMA Netw Open*. 2023;6(7):e2322520. doi:10.1001/jamanetworkopen.2023.22520

eTable. Managed Long-term Care Mandate Rollout Dates

This supplementary material has been provided by the authors to give readers additional information about their work.

© 2023 Harrison JM et al. *JAMA Network Open*.

eTable. Managed Long-term Care Mandate Rollout Dates

Region	Counties in Region	Announcement Letter Date†
1	New York City (Bronx, Kings, New York, Queens, Richmond)	June 2012
2	Nassau, Suffolk, Westchester	January 2013
3	Orange, Rockland	June 2013
4	Albany, Erie, Monroe, Onondaga	December 2013
5	Columbia, Putnam, Sullivan, Ulster	April 2014
6	Cayuga, Herkimer, Oneida, Rensselaer	May 2014
7	Greene, Saratoga, Schenectady, Washington	June 2014
8	Broome, Dutchess, Fulton, Montgomery, Schoharie	August 2014
9	Delaware, Warren	September 2014
10	Madison, Niagara, Oswego	October 2014
11	Chenango, Cortland, Genesee, Livingston, Ontario, Orleans, Otsego, Steuben, Tioga, Tompkins, Wayne, Wyoming	December 2014
12	Cattaraugus	March 2015
13	Allegany, Chautauqua, Chemung, Clinton, Essex, Franklin, Hamilton, Jefferson, Lewis, Schuyler, Seneca, St Lawrence, Yates	June 2015

† For analytic purposes, we used the MLTC announcement letter date as the start date.

© 2023 Harrison JM et al. *JAMA Network Open*.
