# Supplementary material for: Changes in Nursing Home Use Following Medicaid-Supported Expanded Access to Home- and Community-Based Services for Older Adults With Dementia
Source: JAMA Netw Open. 2023 Jul 10;6(7):e2322520. doi: 10.1001/jamanetworkopen.2023.22520 (PMC10334251; doi:10.1001/jamanetworkopen.2023.22520)
Supplement: Supplement 2. — Data Sharing Statement [file jamanetwopen-e2322520-s002.pdf]

Data Sharing Statement

Harrison. Changes in Nursing Home Use Following Medicaid-Supported Expanded Access to Home- and Community-Based Services for Older Adults With Dementia. *JAMA Netw Open*. Published July 10, 2023. doi:10.1001/jamanetworkopen.2023.22520

Data
Data available: No
